# The effects of visual imagery on face identification: an ERP study

**DOI:** 10.3389/fnhum.2012.00305

**Published:** 2012-11-08

**Authors:** Jianhui Wu, Hongxia Duan, Xing Tian, Peipei Wang, Kan Zhang

**Affiliations:** ^1^Key Laboratory of Behavioral Science, Institute of Psychology, Chinese Academy of SciencesBeijing, China; ^2^University of the Chinese Academy of SciencesBeijing, China; ^3^Department of Psychology, New York UniversityNew York, NY, USA; ^4^Center for Higher Brain Functions, Capital Medical UniversityBeijing, China

**Keywords:** visual imagery, face identification, event-related potentials, N170, N2, matching

## Abstract

The present study tested the hypothesis that the effects of mental imagery on subsequent perception occur at a later matching stage in perceptual identification, but not in the early perceptual stage as in perceptual detection. The behavioral results suggested that the effect of visual imagery on visual identification is content-specific, i.e., imagining a congruent face facilitates face identification, whereas a mismatch between imagery and perception leads to an interference effect. More importantly, the ERP results revealed that a more negative N2 response to the subsequent visual face stimuli was elicited over fronto-central sites in the mismatch and no-imagery conditions as compared to that in the match condition, with the early P1 and N170 components independent of manipulations. The latency and distribution of the neural effects demonstrate that the matching step, but not the earlier perceptual process, is affected by the preceding visual imagery in the context of face identification. We discuss these results in a broader context that the imagery-perception interaction may depend on task demand.

## Introduction

Our visual perception is shaped by our previous visual experience in the real world (Gilbert, 1996; Webster and MacLeod, 2011). The visual imagery process simulates perceptual representations on the basis of past experience and provides a mental template that can influence the subsequent perception (Moulton and Kosslyn, 2009). Evidence supporting the modulation effects of mental imagery, however, demonstrates distinct directional influence on perception. Some studies show that imagery interferes with perception (Perky, 1910; Segal and Fusella, 1970; Reeves, 1981; Craver-Lemley and Reeves, 1987; Craver-Lemley et al., 1997; Ishai and Sagi, 1997b; Craver-Lemley and Arterberry, 2001); whereas others show facilitation effects (Freyd and Finke, 1984; Farah, 1985; Ishai and Sagi, 1995, 1997a; Pearson et al., 2008).

Several factors have been proposed to reconcile the conflicting results about the different directional effects of mental imagery. The direction of imagery modulation has been hypothesized to be content-specific. That is, whether preceding imagery facilitates or interferes with perception depends on how similar the imagined and presented patterns are. Subjects were more likely to perceive a stimulus when the imagined content matched the presented stimulus than when the two were mismatched (Peterson and Graham, 1974; Farah, 1985, 1989; Finke, 1986; Farah et al., 1988; Djordjevic et al., 2004a,b).

Task demands can be another factor that determines the direction of interaction between imagery and perception. The interference effect was found when the subsequent perceptual task was a simple detection task, i.e., to give a single response to any stimuli presented while not necessary to identify them; whereas the facilitation effect was observed during an identification task, i.e., to give the appropriate response to different stimuli (Finke, 1986). Detection is modeled as a task that only needs to register the presence of a stimulus in an all-or-none fashion; further processing of any specific features of stimuli are not required, and may even slow down the detection performance. In contrast, identification is modeled as a task that necessitates the processing of detailed featural information for the following matching processing, in which the comparison between the bottom-up sensory information and the top-down formed template is carried out to classify the sensory input. Most of the previous studies focused on the effects of imagery on subsequent perceptual detection, in which both directional modulations were found (e.g., Segal and Fusella, 1970; Farah, 1985). With only a few studies investigating the after-effect on identification (Finke, 1986; McDermott and Roediger, 1994; Cabeza et al., 1997; Michelon and Koenig, 2002), consistent facilitation effects were reported, i.e., mental imagery of a visual pattern in advance will facilitate the identification of the same pattern.

The neural mechanisms mediating the interaction between imagery and perception are also unclear. One of the questions is that at which level the top-down and bottom-up processes interact. The dominant interpretation is the *perceptual level hypothesis* in which this facilitation/interference effect occurs at the early perceptual level, where the visual processing of visual features in external stimuli is directly manipulated by preceding imagery (Peterson and Graham, 1974; Neisser, 1976; Freyd and Finke, 1984; Farah, 1985; Craver-Lemley and Reeves, 1987; Ishai and Sagi, 1995; Pearson et al., 2008). However, the differential observations of interference and facilitation effects during detection and identification tasks (Finke, 1986), lead us to propose, along the line of a similar theory (Finke, 1986), that perceptual task demand may influence the occurrence of imagery-perception interaction at distinct stages along the visual information processing stream. Specifically, the effects of imagery on perceptual identification occur at the later stage where integrated features of an object are matched with those stored in memory to achieve recognition (referred to as the *matching level hypothesis* henceforth in this paper); whereas the effects of imagery on perceptual detection are presented in early perceptual processes where the representation of object features in establishing and spotting the existence of features in an all-or-none fashion would be sufficient in the task of detection *(perceptual level hypothesis)*.

To distinguish the *perceptual level hypothesis* and the *matching level hypothesis*, we need to investigate the dynamics of cognitive functions. It is hard, if not impossible, to separate different cognitive stages using behavioral experiments as in most previous studies, because the behavioral performance is the cumulative result of processing at multiple levels. Moreover, the behavioral measures suffer from methodological limitations, such as confounds from experimenter expectancy effects and subjects' tacit knowledge (Farah et al., 1988; Pylyshyn, 2002). The event-related potentials (ERP) technique, on the other hand, is an objective measure that may be relatively less confounded by these strategic effects. More importantly, with its high temporal resolution, ERP can be used to determine the time course of neural activity, making it possible to determine the cognitive stage at which mental imagery has effects on perception.

To our knowledge, only two ERP studies have been carried out to investigate the effects of visual imagery on perception, using either a detection task paradigm (Farah et al., 1988) or without an active task (Ganis and Schendan, 2008). Farah et al. (1988) found larger early negativity to the subsequent visual stimuli which peaked at 160 ms over temporo-occipital sites when the imagery matched perception compared with the mismatch condition. Consistently, stimuli were detected more often for the match than for the mismatch condition. Ganis and Schendan (2008) observed that both perception and imagery affect the N170 response to the subsequently presented test faces. Specifically, the amplitude of N170 was enhanced when they were preceded by face imagery rather than object imagery, and similar effects were found for the non-face objects. Interestingly, Sreenivasan et al.'s study (2007) also found that noise probes presented during the delay interval of a delayed-recognition task elicited a larger N170 during face relative to house working memory. These ERP experiments provided neural evidence for the *perceptual level hypothesis* for visual detection. In these two ERP experiments, however, no identification task was implemented; it is still unclear at which stage mental imagery affects the identification task.

The goal of the present ERP study was to test the proposed *matching level hypothesis* in visual identification by examining the cognitive stage(s) at which the interaction occurs between face imagery and face identification. We designed a face imagery-face identification paradigm, during which participants were required to imagine one of two faces or without any imagery followed by an identification task to visually presented face pictures. We predicted that, behaviorally, matching between imagined and presented faces will lead to a facilitation effect as compared with the no imagery condition, and mismatch will lead to an interference effect. For the electrophysiological recording, at least two processing components were proposed in the context of face recognition (Bruce and Young, 1986): the early pre-categorical structural encoding of faces reflected in the early N170 component (e.g., Sagiv and Bentin, 2001) and a later matching process between encoded facial representation and stored structural codes (templates) presumably mediated by a fronto-central distributed N2 component that is usually elicited by a perceptual mismatch from a template and detection of novelty (for a review, see Folstein and Van Petten, 2008). Some studies reported that the earlier P1 component is also related to the earlier stage of face perception (Itier and Taylor, 2004; Thierry et al., 2007). According to the *matching level hypothesis*, this N2 component is predicted to be modulated by preceding imagery; whereas the modulation of the P1 and N170 responses is predicted by the *perceptual level hypothesis* and any changes observed in these early components will suggest the formation of perceptual features in the identification task is equally affected by preceding mental imagery.

## Materials and methods

### Participants

Data from 24 participants (mean age 22 ± 1.7 years, 12 men, all right-handed) were collected and analyzed. All were undergraduates from China Agricultural University and Beijing Forestry University who gave informed consent and were paid for their participation. None of them had a history of neurological or psychiatric disorders. All reported normal hearing and normal or corrected-to-normal vision. The experiment had been approved by the Ethics Committee of Human Experimentation in the Institute of Psychology, Chinese Academy of Sciences.

### Stimuli

**Figure 1:**
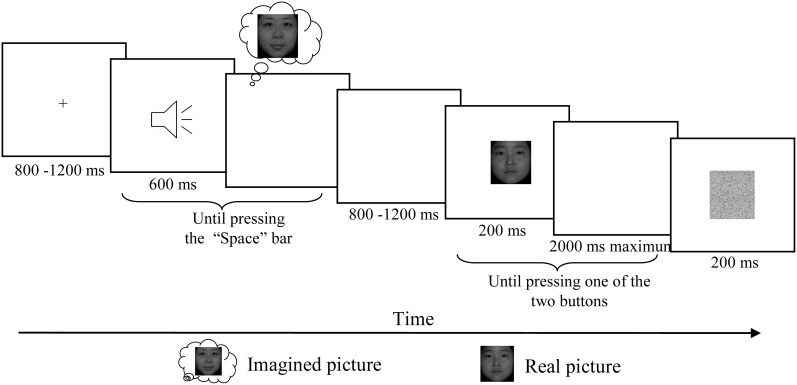
**Schematic description of the experimental paradigm.** After the presentation of fixation, one of these three letter sounds was presented and participants were asked to imagine the corresponding face vividly and then press the “Space” bar in the self-paced interval. For the no imagery associated sound, participants were asked to simply press “Space” once hearing the letter. After a random delay between 800 and 1200 ms, one of these two real faces was presented for 200 ms and participants were asked to press one of the two buttons according to which of the two faces presented on the screen. After the response a visual noise mask with duration of 200 ms was presented before the next trial began.

Two female faces with neutral expression from the Revision of the Chinese Facial Affective Picture System (Gong et al., 2011) were chosen as visual stimuli for both imagery and real presentation. These two pictures and one visual noise mask (Figure 1) were presented on a computer screen placed 75 cm away from the participants' eyes and subtended at an angle of approximately 7° horizontally and 7.7° vertically. Three auditory vowels (a, o, and u) were used as acoustic cues to indicate different tasks (imagery vs. no-imagery) as well as content in the imagery tasks. Specifically, two of them indicated participants to visualize different faces and the third one reminded participants not to imagine anything (no-imagery condition). The associations between auditory cues and imagined faces/no imagery were counterbalanced across participants. The duration of each sound was 600 ms and the intensity was adjusted to a comfortable listening level of about 70 dB SPL using Adobe Audition (version 1.0). Sounds were delivered binaurally through headphones by the Stim interface system (Neuroscan Labs, Sterling, VA).

### Procedure

Participants were seated in a relaxed position on a comfortable chair in a dimly lit, sound-attenuated, and electrically isolated room. Participants completed six testing blocks while EEG was recorded. Each block started with a short familiarization session to remind participants of the pairing of auditory cues and tasks. After a fixation presented with duration between 800 and 1200 ms, one of the auditory cues was presented and the participants were asked to press the “Space” bar after they vividly formed the image of the corresponding face. In the no-imagery condition, participants were asked to press “Space” once hearing the letter. A random delay between 800 and 1200 ms was inserted before a visual face stimulus was presented for 200 ms followed by a maximal 2000 ms response window, in which participants were asked to press the left button for one face and right button for another face as quickly and accurately as possible. A visual noise mask with duration of 200 ms was presented before the next trial began (Figure 1).

This experiment included three conditions that differed in the tasks associated with the different auditory cues. Participants were asked either to skip an auditory cue (no-imagery condition), or to vividly visualize a corresponding face that could be congruent (match condition) or incongruent (mismatch condition) with the subsequent visual stimuli. Sixty testing trials were included in each block, yielding 120 trials for each condition. The two visual face stimuli were presented equally often in each condition. A pseudo-random presentation order was used, so that participants could not predict the upcoming visual face stimuli. Sequential effects of trial-to-trial transitions were also counterbalanced within each block.

Four training sessions were run before the EEG recordings to ensure the correct pairing of auditory cue and imagery, as well as the vividness of visual imagery. The first session was a familiarization session in which the auditory cues and corresponding face pictures (or no picture) were presented simultaneously at least 35 times for each pair, until participants reported that they had learned these associations. The second session was an imagery training session in which only the auditory cues were presented and the participants were encouraged to vividly imagine the corresponding faces; the correct pairing of visual stimulus and auditory cue was then presented, and participants were required to adjust their imagination. For the no imagery condition, only a vowel sound was presented and no imagination or subsequent adjustment was required. Each pair was repeated 20 times to ensure that participants were able to imagine the corresponding faces vividly. The third session was a face identification training session in which one of these two faces was presented and the participants were asked to identify the presented face by pressing the “left” or “right” key as quickly and accurately as possible. The association of visual stimuli and response keys was counterbalanced across participants. Feedback was provided following responses. The fourth session was an imagery-perception training session with identical procedure to the testing blocks, except feedback was provided after their responses instead of a white noise mask. For all the four training sessions, each participant received equal exposure to both faces.

Participants completed a brief questionnaire by rating the vividness of their visual imagery on a 7-point scale (1 = very vivid imagery, 7 = no imagery at all) at the end of this experiment.

### EEG recording and analysis

EEG data were continuously recorded from 64 cap-mounted Ag/AgCl electrodes arranged according to the 10–20 international placement system (Neuroscan Inc.) with an on-line reference to the left mastoid and off-line algebraic re-reference to the average of the left and right mastoids. The EEG data were amplified with a bandpass filter of 0.05–100 Hz and digitized at 500 Hz. The vertical and horizontal electrooculogram (VEOG and HEOG) were recorded from two pairs of electrodes: one pair placed 1 cm above and below the left eye, and another pair placed 1 cm lateral from the outer canthi of both eyes. Interelectrode impedances were maintained below 5 kΩ.

The EEG data were processed offline using the Neuroscan 4.3 software. Ocular artifacts were removed using a regression procedure implemented in the Neuroscan software (Semlitsch et al., 1986). Data were lowpass filtered with cutoff frequency at 30 Hz and epochs of 400 ms in duration (including 100 ms of pre-stimulus time as a baseline) were extracted, time-locked to the onset of visual stimuli. Epochs exceeding ±70 μV were considered artifacts and rejected from further analysis. Average responses were obtained for each condition.

The peak amplitude and latency of P1 were measured at electrodes PO7, CB1, O1 PO8, CB2, and O2 and were subjected to a repeated measures Three-Way ANOVA with factors of matching (mismatch, match and no-imagery) × laterality (left and right) × sites. The mean amplitude of N170 was measured in the time window of 140–180 ms over 12 parieto-occipital sites (P7, PO3, PO5, PO7, O1, CB1, P8, PO4, PO6, PO8, O2, and CB2) and was subjected to a repeated measures Three-Way ANOVA with factors of matching (mismatch, match and no-imagery) × laterality (left and right) × sites. Given that the N170 originates in temporal regions (e.g., Henson et al., 2003), analysis in posterior and inferior temporal channels seems necessary. We thus ran separate ANOVAs for each of the four channels (TP7, TP8, T7, and T8), though the N170 amplitude was small or the polarity was reversed in these channels. The mean amplitude of the N2 component was measured in the time window of 250–350 ms at the following 21 sites: Fz, FCz, Cz, CPz, Pz, POz, Oz, F3, FC3, C3, CP3, P3, PO3, O1, F4, FC4, C4, CP4, P4, PO4, and O2. The N2 amplitudes were subjected to a repeated measures Three-Way ANOVA with factors of matching (mismatch, match and no imagery) × anterior-posterior scalp location (F, FC, C, CP, P, PO, and O) × laterality (left, midline and right). Three additional ANOVAs were carried out to directly test the distinct neural correlates of facilitation/interference with paired matching conditions (match vs. mismatch, match vs. no-imagery, and mismatch vs. no-imagery). Behaviorally incorrect trials were excluded from analysis. The Greenhouse–Geisser correction was used to adjust for sphericity violations. The Bonferroni correction was applied for multiple comparisons. We also performed *post-hoc* Pearson correlation analyses in order to assess the relationship between ERP components and actual behavior (two-tailed).

## Results

### Behavior and post-experimental questionnaire

**Figure 2:**
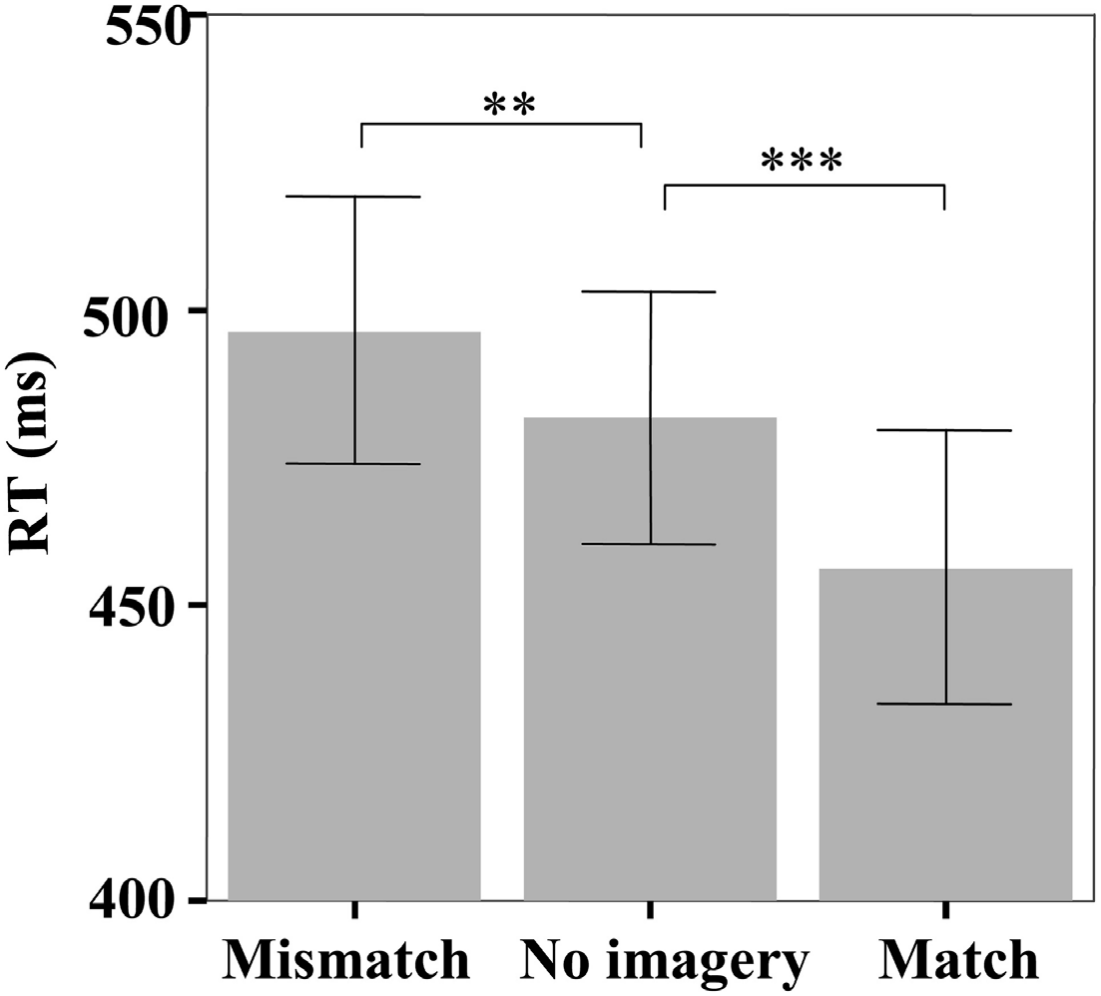
**The mean reaction times in the face identification task.** Error bars represent two standard errors of the mean (SEM). ^**^p < 0.01; ^***^p < 0.001.

The mean reaction time (RT) in the face identification task was significantly different among the three matching conditions [*F*_(2, 46)_ = 26.07, *p* < 0.001] (Figure 2). Pairwise comparisons indicated that participants reliably responded faster on match trials than both no imagery trials (456 vs. 482 ms, *p* < 0.001) and mismatch trials (456 vs. 497 ms, *p* < 0.001), but slower on mismatch trials than on no imagery trials (497 vs. 482 ms, *p* = 0.01). The response accuracy in the face identification task was not significantly different among the three matching conditions [*F*_(2, 46)_ = 1.88, *p* = 0.17; mismatch: 95.58%, match: 97.02%, no-imagery: 96.44%].

The mean RT of “Space” bar presses after the onset of the auditory cue was significantly different among the three cues [*F*_(2, 46)_ = 8.50, *p* = 0.001]. Pairwise comparisons indicated that participants spent less time after the no-imagery cue than after the two face-imagery cues (976 ± 265 ms vs. 1358 ± 698 ms, *p* < 0.01 and 976 ± 265 ms vs. 1359 ± 806 ms, *p* < 0.05), but there was no significant difference between the two face-imagery cues (*p* = 0.98). The post-experimental questionnaire revealed that participants had experienced vivid face imagery (2.54 ± 0.72) when the imagery cues were presented.

### ERP results

**Figure 3:**
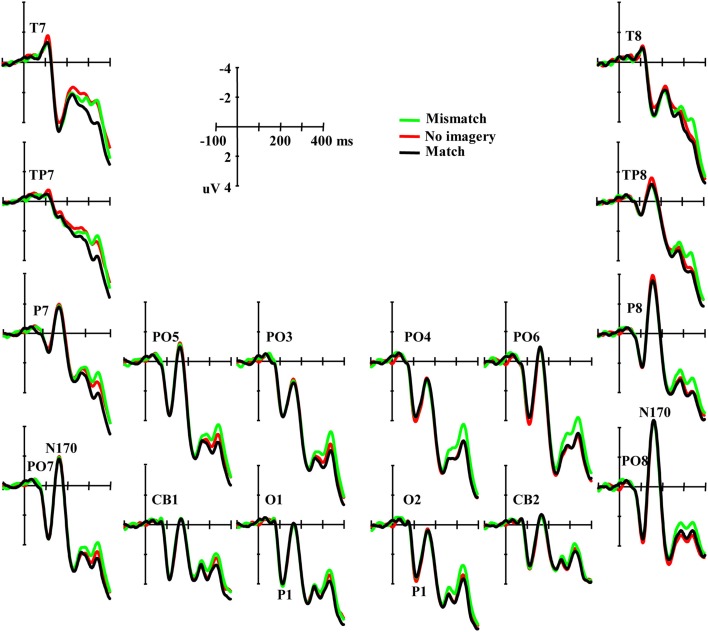
**Grand averaged ERPs illustrating the P1 and the N170 elicited by visual stimuli in three conditions**.

An occipital P1 was observed in all three conditions (Figure 3). ANOVA did not reveal any significant difference among conditions for either the peak amplitude or latency of the P1 component [*F*_(2, 46)_ = 0.42, *p* = 0.66 and *F*_(2, 46)_ = 1.62, *p* = 0.21, respectively], and also no interaction effect between matching conditions and laterality [*F*_(2, 46)_ = 0.57, *p* = 0.57 and *F*_(2, 46)_ = 1.18, *p* = 0.31, ε = 0.72, respectively]. Additional correlation analysis revealed no significant correlation between RT change and the P1 amplitude/latency differences between the paired comparisons of the three conditions (*P*s > 0.10).

Typical N170 potentials were observed in responses to visual face stimuli over parieto-occipital sites (Figure 3). ANOVA did not reveal any significant difference among conditions for the mean amplitude of the N170 component [*F*_(2, 46)_ = 0.091, *p* = 0.91], and also no interaction effect between matching conditions and laterality or sites [*F*_(2, 46)_ = 0.70, *p* = 0.50 and *F*_(10, 230)_ = 1.01, *p* = 0.42, ε = 0.60, respectively]. Additional correlation analysis revealed no significant correlation between RT change and the N170 amplitude differences across all the 12 channels between the paired comparisons of the three conditions (*P*s > 0.10). ANOVAs also did not reveal any significant differences among conditions in TP7, TP8, T7, and T8 (*P*s > 0.10).

**Figure 4:**
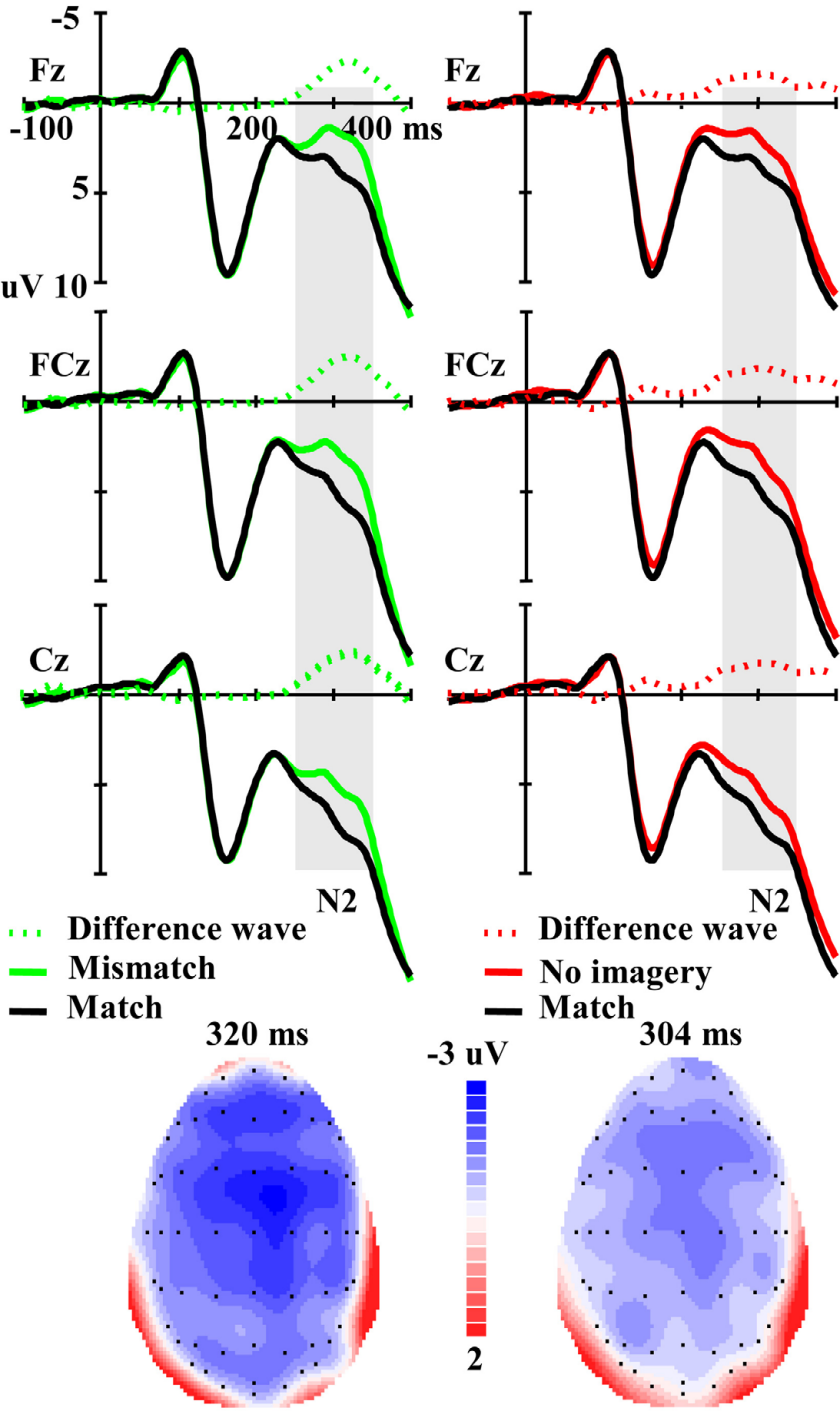
**The left panel illustrates the grand averaged ERPs in mismatch and match conditions and their difference wave. The right panel illustrates the grand averaged ERPs in no imagery and match conditions and their difference wave.** The gray areas highlight the time windows of N2 (250–350 ms) used for statistical analysis. The scalp distributions are time-locked to the peak amplitude of the difference wave (Left: Mismatch minus Match; Right: No imagery minus Match).

For the amplitude of the N2 component elicited by real faces (Figure 4), ANOVA revealed a significant main effect of matching (mismatch, match and no-imagery) [*F*_(2, 46)_ = 5.56, *p* < 0.01], and a marginally significant interaction effect between matching factors and anterior-posterior scalp location [*F*_(12, 276)_ = 2.50, *p* < 0.10, ε = 0.21]. One additional ANOVA indicated a significant main effect of matching (mismatch vs. match) [*F*_(1, 23)_ = 13.92, *p* < 0.01] and an interaction effect between matching (mismatch vs. match) and anterior-posterior electrodes [*F*_(6, 138)_ = 3.39, *p* < 0.05, ε = 0.29]. *Post-hoc* analyses revealed that the mismatch condition was more negative than the match condition and this effect was broadly distributed along the anterior–posterior dimension (*P*s < 0.01), but was maximal at the fronto-central areas. Correlation analysis revealed a marginally significant negative correlation between RT change (mismatch vs. match) and the N2 amplitude change (mismatch-match) over anterior–posterior sensors (*r* = −0.347, *p* = 0.096) (i.e., the longer the RT change, the larger the N2 amplitude change). Another additional ANOVA indicated a significant main effect of matching (no-imagery vs. match) [*F*_(1, 23)_ = 4.60, *p* < 0.05] and an interaction effect between matching (no-imagery vs. match) and anterior-posterior electrodes [*F*_(6, 138)_ = 4.47, *p* < 0.05, ε = 0.27]. *Post-hoc* analyses revealed that the no-imagery condition was more negative than the match condition and this effect was broadly distributed along the fronto–parietal areas (*P*s < 0.05), and was maximal at the fronto-central areas, but not at the parieto-occipital and occipital areas (*p* = 0.36 and 0.52, respectively). Correlation analysis revealed a significant negative correlation between RT change (no imagery vs. match) and the N2 amplitude change (no imagery-match) over fronto–parietal sensors (*r* = −0.422, *p* < 0.05). There was no significant difference between mismatch and no imagery conditions (*p* = 0.39) and no interaction effect between imagery (mismatch and no imagery) and anterior-posterior electrodes (*p* = 0.55).

## Discussion

The present study investigated the effects of mental imagery on subsequent face identification. Behaviorally, participants responded faster in the match condition than in the no-imagery condition, with the slowest RT in the mismatch condition, which provides further evidence supporting the hypothesis that the imagery-perception interaction is content-specific in an identification task. In our experiment, we demonstrated the content-specific effects extending to complex visual stimuli, human faces in this case, compared to the simple visual objects and features used previously (Finke, 1986). The results of the imagery vividness questionnaire suggest that the subjects successfully executed the visual imagery. Participants pressed the “Space” bar more rapidly to the no-imagery cue than to face-imagery cues, providing further evidence for the execution of the visual imagery task as required.

Consistent with these behavioral measures, ERP results showed that, compared with the match condition, both mismatch and no-imagery conditions elicited a higher amplitude of the fronto-central maximal N2 (250–350 ms window). No significant difference was observed for the P1 and N170 components. The observation of changes in the later N2 component but not in early visual processing components provides neural evidence supporting the *matching level hypothesis*: imagined visual stimuli affected the matching stage, rather than the earlier perceptual processing stages in the visual identification task. Pearson correlation analyses revealed that the longer the RT change, the larger the N2 amplitude change from match to mismatch/no imagery condition, but not for P1 or N170 amplitude change, providing further evidence for the *matching level hypothesis*.

The mismatch condition elicited higher amplitude of the fronto-central maximal N2 during the 250–350 ms window, and this result is consistent with previous findings using repetition paradigms. The fronto-central N2 component at this time range is usually elicited under the S1–S2 matching task, where the S1 can be presented physically or formed from imagery (Wu et al., 2010). This component has been generally considered to be sensitive to mismatch from visual templates (for a review, see Folstein and Van Petten, 2008). Wang et al. (2001) have revealed that the mismatch-related N2 was elicited independently of task demands. Results from studies using repetition paradigms also revealed that, compared with the primed target, the unprimed target (mismatch) elicited a more negative anterior-distributed negativity around 250–350 ms after stimulus onset (Eddy et al., 2006; Eddy and Holcomb, 2011). According to the model of face recognition (Bruce and Young, 1986), a sense of familiarity is achieved, if the bottom-up sensory process matches the stored structure code retrieved from memory. The mental imagery of a face, in this study, is a top-down process that forms an internal perceptual template of a face representation (Kosslyn et al., 2006; Wu et al., 2011), and this perceptual template provides a recent context for the subsequent visual target. When the features of visual stimuli match the pre-activated face template from preceding mental imagery, the identification can be achieved faster, resulting in the behavioral benefits in the matching condition. Whereas when the internal image is incompatible with the target, as in the mismatch condition, more time and higher neural cost is required to resolve the conflict, manifested in a behavioral interference effect and larger N2 amplitude.

Our ERP results also revealed that the fronto-central distributed N2 was observed in the no-imagery condition. There might be two cognitive mechanisms for this result, and both of them can be considered occurring at the matching stage of face identification. The first one is the mismatch interpretation of this N2 (for a review, see Folstein and Van Petten, 2008). Theories have been proposed that our perception of external objects and events is the result of bottom-up process meeting the consistent top-down prediction (Hochstein and Ahissar, 2002). That is, the automatic predictive process is also available in the no-imagery condition but provides less specific predictions for the coming stimuli compared with the matching imagery. Such under-specified predictions would induce greater mismatches with the bottom-up process and hence elicit larger fronto-central N2 and longer RT. The second one is to explain this N2 as the effect of immediate context. Besides the mismatch interpretation, the fronto-central N2 has also been interpreted as a mechanism of novelty detection (for a review, see Folstein and Van Petten, 2008). Previous studies suggested that both long-term context and immediate context contribute to the novelty response (Daffner et al., 2000a,b). In the current study, the preceding imagery process of match trials provided immediate context for the subsequently presented face of the same trial, thus the presented face is relatively more novel in no-imagery trials than in the match trials; this novelty effect within the trial or in a short-term/immediate context elicited the N2 in the no-imagery condition. The matching stage of face identification in no-imagery trials has to depend on the mechanism of the long-term memory system (Grützner et al., 2010), then more time and higher neural cost is required as compared with that in the match condition during which the short-term memory trace can be accessed from preceding mental imagery.

It might be argued that the difference between conditions can also be interpreted as an Late Positive Complex (LPC) effect but not the N2 effect, i.e., the match condition elicited more positive potentials than both the mismatch and no-imagery conditions. The fronto-central LPC has been related to the old/new effect where old stimuli elicit more positive LPC than do new stimuli, and further studies suggested that this fronto-central old-new effect was an index of familiarity (for a review, see Rugg and Curran, 2007). In the current study, we interpreted that the no imagery condition was relatively more novel in a short-term context, and thus elicited more negative fronto-central N2 than the match condition and we interpreted it as a mechanism of novelty detection, i.e., novel faces elicit more negative N2. Thus, the trend between conditions was the same for both components/interpretations. More importantly, both components have the same scalp distribution and similar time window. Thus, we argue that both components and interpretations are not contrary and may in fact reflect similar cognitive processing. For the difference between match and mismatch conditions, we focus on the mismatch interpretation because the current experimental design is quite different from that used in research on the old/new effect.

The distinct temporal profiles of neural correlates suggest that task demand may be an important factor determining the imagery-perception interaction in the hierarchical visual process. The present study revealed that the effects of mental imagery on visual identification occur at a later matching stage, where feature information from bottom-up processes is matched with that from top-down processes to achieve recognition. Thus the *matching level hypothesis* is supported in a visual identification task. Previous ERP studies provided neural evidence that, during a detection task or without an active task, imagery-perception interaction occurs at the earlier perceptual stage of visual processing, thus supporting the *perceptual level hypothesis* (Farah et al., 1988; Ganis and Schendan, 2008). Task demand modulates not only the direction that imagery affects perception (Finke, 1986) but also the cognitive stage(s) at which this influence occurs.

Some limitations should be noted. The first limitation is that only face stimuli were used in this study. A previous study by Ganis and Schendan (2008) demonstrated that face imagery affected the early perceptual processing of subsequent test faces (no task on the test faces) and such effects could generalize to other object categories. The choice of only including face stimuli in our study is because of the emphasis on testing the perceptual and matching level hypotheses in the context of face identification. But without stimuli from other categories as comparison conditions, we cannot generalize the observed effects of visual imagery to other perceptual categories. The second limitation is that only two tokens of face stimuli were used in this study. This choice is because, arguably, fewer tokens to imagine would result in more vivid visual reconstructions after equal amounts of training, and hence increase the effect sizes of mental imagery on subsequent perception. However, participants could adapt strategies by visualizing either specific parts/features or the global configuration of a face, thus it is not clear which processing mechanisms would account for the observed effects of mental imagery on visual face perception. The third limitation is that the 100 ms pre-face stimulus baseline might bring in inequity between the imagery and no-imagery conditions. Although in both the current study and previous studies (Farah et al., 1988; Ganis and Schendan, 2008) participants were asked to press a button to separate the mental imagery processing period and the following perception period, participants could inertially perform visual imagery and the visual trace was still available during the pre-face stimulus interval for the imagery conditions, but not for the no-imagery condition. Such potential overlap in the imagery conditions may influence the ERP baseline and result in an amplitude shift in the ERPs to the subsequent face stimuli.

In conclusion, the effect of mental imagery on subsequent face identification is content-specific, i.e., mismatch between the presented and imagined face leads to an interference and match leads to a facilitation effect. The ERP results suggested that both facilitation and interference effects in a face identification task occur at a later matching stage, but not in the early perceptual processing.

### Conflict of interest statement

The authors declare that the research was conducted in the absence of any commercial or financial relationships that could be construed as a potential conflict of interest.

## References

[B1] Bruce V. Young A. (1986). Understanding face recognition. Br. J. Psychol. 77, 305–327 3756376 10.1111/j.2044-8295.1986.tb02199.x

[B2] Cabeza R. Burton A. M. Kelly S. W. Akamatsu S. (1997). Investigating the relation between imagery and perception: evidence from face priming. Q. J. Exp. Psychol. A 50, 274–289 10.1080/027249897392099 9225624

[B3] Craver-Lemley C. Arterberry M. E. (2001). Imagery-induced interference on a visual detection task. Spat. Vis. 14, 101–119 10.1163/156856801300202887 11450798

[B4] Craver-Lemley C. Arterberry M. E. Reeves A. (1997). Effects of imagery on vernier acuity under conditions of induced depth. J. Exp. Psychol. Hum. Percept. Perform. 23, 3–13 9157187 10.1037//0096-1523.23.1.3

[B5] Craver-Lemley C. Reeves A. (1987). Visual imagery selectively reduces vernier acuity. Perception 16, 599–614 3451189 10.1068/p160599

[B6] Daffner K. R. Mesulam M. M. Scinto L. F. M. Calvo V. Faust R. Holcomb P. J. (2000a). An electrophysiological index of stimulus unfamiliarity. Psychophysiology 37, 737–747 10.1111/1469-8986.3760737 11117454

[B7] Daffner K. R. Scinto L. F. M. Calvo V. Faust R. Mesulam M. M. West W. C. (2000b). The influence of stimulus deviance on electrophysiologic and behavioral responses to novel events. J. Cogn. Neurosci.12, 393–406 10931766 10.1162/089892900562219

[B8] Djordjevic J. Zatorre R. J. Jones-Gotman M. (2004a). Effects of perceived and imagined odors on taste detection. Chem. Senses 29, 199–208 10.1093/chemse/bjh022 15047594

[B9] Djordjevic J. Zatorre R. J. Petrides M. Jones-Gotman M. (2004b). The mind's nose: effects of odor and visual imagery on odor detection. Psychol. Sci. 15, 143–148 15016284 10.1111/j.0956-7976.2004.01503001.x

[B10] Eddy M. D. Holcomb P. J. (2011). Invariance to rotation in depth measured by masked repetition priming is dependent on prime duration. Brain Res. 1424, 38–52 10.1016/j.brainres.2011.09.036 22005687 PMC3218085

[B11] Eddy M. Schmid A. Holcomb P. J. (2006). Masked repetition priming and event-related brain potentials: a new approach for tracking the time-course of object perception. Psychophysiology 43, 564–568 10.1111/j.1469-8986.2006.00455.x 17076812 PMC1857300

[B12] Farah M. J. (1985). Psychophysical evidence for a shared representational medium for mental images and percepts. J. Exp. Psychol. Gen. 114, 91–103 3156947 10.1037//0096-3445.114.1.91

[B13] Farah M. J. (1989). Mechanisms of imagery-perception interaction. J. Exp. Psychol. Hum. Percept. Perform. 15, 203–211 2525596 10.1037//0096-1523.15.2.203

[B14] Farah M. J. Peronnet F. Gonon M. A. Giard M. H. (1988). Electrophysiological evidence for a shared representational medium for visual images and visual percepts. J. Exp. Psychol. Gen. 117, 248–257 2971761 10.1037//0096-3445.117.3.248

[B15] Finke R. A. (1986). Some consequences of visualization in pattern identification and detection. Am. J. Psychol. 99, 257–274 3766817

[B16] Folstein J. R. Van Petten C. (2008). Influence of cognitive control and mismatch on the N2 component of the ERP: a review. Psychophysiology 45, 152–170 10.1111/j.1469-8986.2007.00602.x 17850238 PMC2365910

[B17] Freyd J. J. Finke R. A. (1984). Facilitation of length discrimination using real and imagined context frames. Am. J. Psychol. 97, 323–341 6496807

[B18] Ganis G. Schendan H. E. (2008). Visual mental imagery and perception produce opposite adaptation effects on early brain potentials. Neuroimage 42, 1714–1727 10.1016/j.neuroimage.2008.07.004 18674625

[B19] Gilbert C. D. (1996). Plasticity in visual perception and physiology. Curr. Opin. Neurobiol. 6, 269–274 10.1016/S0959-4388(96)80083-3 8725971

[B20] Gong X. Huang Y. X. Wang Y. Luo Y. J. (2011). Revision of the Chinese facial affective picture system. Chin. Ment. Heal. J. 25, 40–46

[B21] Grützner C. Uhlhaas P. J. Genc E. Kohler A. Singer W. Wibral M. (2010). Neuroelectromagnetic correlates of perceptual closure processes. J. Neurosci. 30, 8342–8352 10.1523/JNEUROSCI.5434-09.2010 20554885 PMC6634569

[B22] Henson R. N. Goshen-Gottstein Y. Ganel T. Otten L. J. Quayle A. Rugg M. D. (2003). Electrophysiological and haemodynamic correlates of face perception, recognition and priming. Cereb. Cortex 13, 793–805 10.1093/cercor/13.7.793 12816895

[B23] Hochstein S. Ahissar M. (2002). View from the top: hierarchies and reverse hierarchies in the visual system. Neuron 36, 791–804 10.1016/S0896-6273(02)01091-7 12467584

[B24] Ishai A. Sagi D. (1995). Common mechanisms of visual imagery and perception. Science 268, 1772–1774 10.1126/science.7792605 7792605

[B25] Ishai A. Sagi D. (1997a). Visual imagery facilitates visual perception: psychophysical evidence. J. Cogn. Neurosci. 9, 476–489 10.1162/jocn.1997.9.4.476 23968212

[B26] Ishai A. Sagi D. (1997b). Visual imagery: effects of short- and long-term memory. J. Cogn. Neurosci. 9, 734–742 10.1162/jocn.1997.9.6.734 23964596

[B27] Itier R. J. Taylor M. J. (2004). N170 or N1? Spatiotemporal differences between object and face processing using ERPs. Cereb. Cortex 14, 132–142 10.1093/cercor/bhg111 14704210

[B28] Kosslyn S. M. Thompson W. L. Ganis G. (2006). The Case for Mental Imagery, New York, NY: Oxford University Press

[B29] McDermott K. B. Roediger H. L. 3rd. (1994). Effects of imagery on perceptual implicit memory tests. J. Exp. Psychol. Learn. Mem. Cogn. 20, 1379–1390 7983469 10.1037//0278-7393.20.6.1379

[B30] Michelon P. Koenig O. (2002). On the relationship between visual imagery and visual perception: evidence from priming studies. Eur. J. Cogn. Psychol. 14, 161–184

[B31] Moulton S. T. Kosslyn S. M. (2009). Imagining predictions: mental imagery as mental emulation. Philos. Trans. R. Soc. Lond. B Biol. Sci. 364, 1273–1280 10.1098/rstb.2008.0314 19528008 PMC2666712

[B32] Neisser U. (1976). Cognition and Reality. San Francisco, CA: W. H. Freeman 10.1037/a0029351

[B33] Pearson J. Clifford C. W. Tong F. (2008). The functional impact of mental imagery on conscious perception. Curr. Biol. 18, 982–986 10.1016/j.cub.2008.05.048 18583132 PMC2519957

[B34] Perky C. W. (1910). An experimental study of imagination. Am. J. Psychol. 21, 422–452

[B35] Peterson M. J. Graham S. E. (1974). Visual detection and visual imagery. J. Exp. Psychol. 103, 509–514 4448955 10.1037/h0037150

[B36] Pylyshyn Z. W. (2002). Mental imagery: in search of a theory. Behav. Brain Sci. 25, 157–182 12744144 10.1017/s0140525x02000043

[B37] Reeves A. (1981). Visual-imagery lowers sensitivity to hue-varying, but not to luminance-varying, visual stimuli. Percept. Psychophys. 29, 247–250 7267276 10.3758/bf03207291

[B38] Rugg M. D. Curran T. (2007). Event-related potentials and recognition memory. Trends Cogn. Sci. 11, 251–257 10.1016/j.tics.2007.04.004 17481940

[B39] Sagiv N. Bentin S. (2001). Structural encoding of human and schematic faces: holistic and part-based processes. J. Cogn. Neurosci. 13, 937–951 10.1162/089892901753165854 11595097

[B40] Segal S. J. Fusella V. (1970). Influence of imaged pictures and sounds on detection of visual and auditory signals. J. Exp. Psychol. 83, 458–464 5480913 10.1037/h0028840

[B41] Semlitsch H. V. Anderer P. Schuster P. Presslich O. (1986). A solution for reliable and valid reduction of ocular artifacts applied to the P300 ERP. Psychophysiology 23, 695–703 3823345 10.1111/j.1469-8986.1986.tb00696.x

[B42] Sreenivasan K. K. Katz J. Jha A. P. (2007). Temporal characteristics of top-down modulations during working memory maintenance: an event-related potential study of the N170 component. J. Cogn. Neurosci. 19, 1836–1844 10.1162/jocn.2007.19.11.1836 17958486

[B43] Thierry G. Martin C. D. Downing P. Pegna A. J. (2007). Controlling for interstimulus perceptual variance abolishes N170 face selectivity. Nat. Neurosci.10, 505–511 10.1038/nn1864 17334361

[B44] Wang H. Wang Y. Kong J. Cui L. Tian S. (2001). Enhancement of conflict processing activity in human brain under task relevant condition. Neurosci. Lett. 298, 155–158 10.1007/s10571-007-9195-4 11165430

[B45] Webster M. A. MacLeod D. I. (2011). Visual adaptation and face perception. Philos. Trans. R. Soc. Lond. B Biol. Sci. 366, 1702–1725 21536555 10.1098/rstb.2010.0360 PMC3130378

[B46] Wu J. Mai X. Yu Z. Qin S. Luo Y. J. (2010). Effects of discrepancies between imagined and perceived sound on the N2 component of the event-related potential. Psychophysiology 47, 289–298 10.1111/j.1469-8986.2009.00936.x 20003146

[B47] Wu J. Yu Z. Mai X. Wei J. Luo Y. J. (2011). Pitch and loudness information encoded in auditory imagery as revealed by event-related potentials. Psychophysiology 48, 415–419 10.1111/j.1469-8986.2010.01070.x 20636291

